# Characteristics of outpatients with functional somatic syndromes at a university hospital's general medicine clinic

**DOI:** 10.1002/jgf2.543

**Published:** 2022-04-05

**Authors:** Junji Nishiyama, Tetsuya Abe, Sumito Imaizumi, Akira Yamane, Mikihiko Fukunaga

**Affiliations:** ^1^ Department of Psychosomatic and General Internal Medicine Kansai Medical University Hirakata Japan; ^2^ Clinic of General Medicine Kansai Medical University Hospital Hirakata Japan

**Keywords:** functional somatic syndromes, general medicine, health‐related quality of life, medically unexplained symptoms, primary care, psychobehavioral factors

## Abstract

**Background:**

The term medically unexplained symptoms (MUS) is unhelpful for both patients and physicians, and more acceptable illness categories are needed as substitutes for MUS. While some potential substitutes are characterized by excessive psychological burden related to somatic symptoms, “functional somatic syndromes” (FSS) is a category that focuses on physical dysfunction and emphasizes similarities among individual syndromes. Examples of FSS include irritable bowel syndrome, functional dyspepsia, and fibromyalgia syndrome. This study aimed to distinguish FSS from MUS and compare the somatic and psychobehavioral characteristics of FSS with those of other diseases.

**Methods:**

This study included 1975 first‐visit outpatients at a Japanese university hospital's general medicine clinic. According to their first‐listed diagnosis, they were classified as having FSS, acute infection, organic disease (OD), psychiatric disorder, and unknown condition (UC). The somatic symptom burden and health‐related quality of life (HRQoL) were assessed using the Somatic Symptom Scale‐8 and EuroQol‐5 Dimension, respectively; the involvement of psychobehavioral factors affecting somatic symptoms was also evaluated.

**Results:**

Overall, 33% of patients were included in the FSS category, and 93% of the supposed MUS (FSS and UC) were diagnosed with FSS. Compared with OD, FSS showed more severe somatic symptom burden, similar reduced HRQoL, and higher involvement of psychobehavioral factors.

**Conclusion:**

It can be useful to improve FSS diagnostic skills for the reduction of MUS misdiagnosis. Psychobehavioral factors might be less associated with MUS (in the narrow sense of the term) than FSS.

## Abstract 2

We classified 1975 first‐visit outpatients at a Japanese university hospital's general medicine clinic as having functional somatic syndrome (FSS), acute infection, organic disease, psychiatric disorder, and unknown condition (UC). FSS comprised one‐third of our study population, and almost all of the supposed medically unexplained symptoms (FSS and UC) were diagnosed with FSS. Compared with other organic diseases, FSS showed more severe somatic symptom burden, similar health‐related quality of life, and higher involvement of psychobehavioral factors.

## 1 INTRODUCTION

There are patients in whom histological and organic disorders cannot be identified based on somatic symptoms in both primary and secondary care settings. Their conditions are recorded as medically unexplained symptoms (MUS),^1^ and medical treatment of such patients places a considerable burden on physicians.^2^ As the term “MUS” can cause an unhelpful explanatory gap between patients and physicians,^3^ more acceptable illness categories are needed. For example, bodily distress disorder^4^ and somatic symptom disorder (SSD)^5^ are illness categories characterized by excessive psychological burden relating to somatic symptoms and are candidates for MUS substitutes.

It is difficult to identify whether patients' functional physical symptoms are rooted in somatic diseases or psychiatric disorders,^6^ and patients exhibiting such symptoms are often misdiagnosed with MUS. FSS refers to several related syndromes that are characterized based on symptoms, suffering, and disability rather than consistently demonstrable tissue abnormalities.^7^ It is a category focused on physical dysfunction, and it emphasizes that similarities among the individual syndromes outweigh the differences. These syndromes include irritable bowel syndrome (IBS), functional dyspepsia, fibromyalgia syndrome (FMS), and chronic fatigue syndrome. Moreover, Wessely et al. have suggested many other syndromes and symptoms, such as premenstrual syndrome and tension headache, for inclusion as FSS.^8^ In our previous studies, we reported lower health‐related quality of life (HRQoL) associated with autonomic dysfunction^9^ and improvement in somatic symptoms and mood disturbance through autogenic training^10^ in patients with FSS. As it is usually less problematic for physicians to assess patients' physical dysfunction than their psychological conditions, the classification of FSS rather than MUS is expected to minimize misdiagnosis. However, the prevalence of FSS in MUS is uncertain.

Most patients with FSS receive unnecessary tests and unfounded reassurance that the disease is not serious and are likely to repeatedly consult other physicians.^11^, ^12^ As a result, the cost of treating such patients becomes a major societal economic issue.^13^ Given that illness behavior depends on the healthcare system and culture of a country,^1^ it is necessary to investigate the behavior in each healthcare system. In systems wherein patients must first visit family physicians for referrals, MUS accounts for 5%–30% of primary care patient diagnoses^2^ and 37%–68% of specialty care patient diagnoses.^14^, ^15^ On the contrary, Japan has a “free access” system wherein patients can utilize whatever kinds of outpatient services they like without referrals. As patients who seek both primary and specialty care can visit university hospitals, epidemiological investigation at a general medicine clinic of a university hospital is suitable to grasp the actual incidence of MUS in Japan. Therefore, this study aimed to identify patients with FSS from patients with MUS and compare their somatic and psychobehavioral characteristics with those of other diseases diagnosed at a university hospital's general medicine clinic.

## 2 METHODS

### 2.1 Design

This was a cross‐sectional survey of outpatients with FSS in the secondary healthcare area in Japan. Before examination, the outpatients who presented at a university hospital's general medicine clinic for the first time were required to complete two questionnaires related to somatic symptom burden and HRQoL. After the examination was complete, the attending physician decided on a diagnosis and the involvement of psychobehavioral factors contributing to the condition.

### 2.2 Participants

The participants in this study were outpatients who presented for the first time at a general medicine clinic of a university hospital in an urban city in western Japan between January 1 and December 31, 2016. Those who were examined by residents and did not complete the questionnaire before examination were excluded from the study. The study protocol was approved by the Institutional Review Board of the university hospital. However, informed consent was not obtained because this study neither involved patients' interventions nor used samples obtained from patients. An opt‐out method was used so that patients could refuse to participate in the study.

### 2.3 Measures

#### 2.3.1 Somatic symptom burden

The Japanese version of the Somatic Symptom Scale‐8 (SSS‐8) is a self‐administered questionnaire that assesses somatic symptom burden;^16^, ^17^ it was originally developed as a shorter version of the Patient Health Questionnaire‐15,^18^ and comprised eight items pertaining to the following categories: (1) stomach or bowel problems; (2) back pain; (3) pain in the arms, legs, or joints; (4) headaches; (5) chest pain or shortness of breath; (6) dizziness; (7) feeling tired or having low energy; and (8) having trouble sleeping. Each item is scored 0 (not at all) to 4 (very much).

#### 2.3.2 HRQoL

The Japanese version of the EuroQol‐5 Dimension (EQ‐5D) has been approved by the EuroQol Group as a concise generic health status instrument that can be used to measure, compare, and value health status across diseases;^19^, ^20^, ^21^ it assesses self‐reported health status across five dimensions (mobility, self‐care, usual activities, pain/discomfort, and anxiety/depression) and three levels of severity (no problems, moderate problems, and severe problems). The responses for the five dimensions were combined into a five‐digit number and were converted to a HRQoL score using Japanese value sets.

### 2.4 Assessment

As of 2016, healthcare insurance at Japanese medical institutions has been organized using diagnostic conditions based on the 10th revision of the International Classification of Diseases (ICD‐10) codes. Therefore, the attending physician decided upon the appropriate diagnostic condition code.

In addition, the condition was classified according to the involvement of psychobehavioral factors using diagnostic criterion B in the Diagnostic and Statistical Manual of Mental Disorders (DSM‐5),^5^ which relates to “psychological factors affecting other medical conditions (316)” (described below):

B. Psychological or behavioral factors adversely affect the medical condition in one of the following ways:

1. These factors influence the course of medical conditions, as demonstrated by the strong temporal association between psychological factors and the development and exacerbation of or delayed recovery from medical conditions.
2. These factors can interfere with the treatment of medical conditions (e.g., poor adherence).
3. These factors are associated with additional well‐established health risks.
4. They can influence the underlying pathophysiology or precipitate and exacerbate symptoms, thereby necessitating medical attention.

### 2.5 Classification

For each patient, the first‐listed diagnosis at initial examination was classified (based on ICD‐10 codes) as follows: FSS, acute infection (AI), organic disease (OD), psychiatric disorder (PSYD), and unknown condition (UC). Patients were considered to have FSS when their condition was one of the conditions proposed by Wessely et al.^8^ or when they had somatic symptoms that met the relevant definitions^7^ and might involve functional pathologies. As infectious diseases are less affected by psychobehavioral factors, we separated AI from other OD. The OD category included any somatic diseases other than AI for which a definitive, organic, or histological diagnosis was made. PSYD included all diagnosed psychiatric disorders except SSD (300.82) and “psychological factors affecting other medical conditions (316),” as defined in the 5th edition of the DSM‐5.^5^ Conditions that were considered not to have a functional pathology at the initial consultation and could not be classified otherwise were defined as UC.

It was not entirely clear which conditions should be classified as functional pathologies because of their variability and instability; therefore, there could be potential discrepancies depending on the individual performing the classification. To address this, classification was performed independently by three coding physicians; two of them were employees at the general medicine clinic, and one was a psychosomatic medicine specialist. Whenever the classification by all the three physicians was not consistent, the final classification was made after discussion among them.

### 2.6 Statistical analysis

Data are presented as mean ± standard deviation. Differences between diagnostic categories in terms of age and somatic symptom burden using the SSS‐8 were assessed using unpaired one‐way analysis of variance. Differences in the HRQoL status between diagnostic categories were assessed using the nonparametric Kruskal–Wallis test, with EQ‐5D utility values. Differences in the involvement of psychobehavioral factors among the five diagnostic categories were assessed using the chi‐squared test. For all the differences identified, multiple comparisons were made using Bonferroni's method. All statistical analyses were performed using SPSS version 26.0 (SPSS Inc.). For all statistical tests, the α level was set at 0.05.

## 3 RESULTS

### 3.1 Participants

A total of 2366 patients presented at a general medicine clinic of a university hospital, but 335 patients who were examined by residents and 56 patients who did not complete the questionnaire before examination were excluded from the study. As a result, 1975 patients were included in the final analysis. The patient population comprised 1239 women (63%) and 736 men. The mean (± standard deviation) age of the patients was 52.6 ± 19.8 years. Overall, 1287 (65%) patients had to pay a supplementary fee of ¥5000 ($47) because they wished to consult a general physician at the university hospital without a referral letter, while 688 (35%) patients were referred to our clinic either from within the hospital or from primary or secondary healthcare centers owing to the nature of their symptoms (unknown cause).

### 3.2 Classification

The proportion (number) of patients classified in each diagnostic category was as follows: FSS, 33% (653); AI, 15% (306); OD, 45% (881); PSYD, 5% (86); and UC, 2.5% (49). Age differences were noted between these categories [F (4, 1970) = 18.160; *p* < 0.001], in that patients in the AI category were younger than those in the other categories, and patients in the FSS category were younger than those in the OD category. No gender differences were noted between the categories (Table 1). AI was mostly reported in the respiratory, urinary, or gastrointestinal tracts.

**TABLE 1:** Characteristics of diagnostic categories

Category	FSS	AI	OD	PSYD	UC	One‐way ANOVA
Number (%)	653 (33.1)	306 (15.5)	881 (44.6)	86 (4.4)	49 (2.5)	
Gender [% female]	65.7	67.3	59.4	62.8	55.1	
Age [Mean ± SD]	51.72 ± 19.96	44.95 ± 18.38	55.75 ± 19.35	52.97 ± 20.39	54.76 ± 18.09	*F*(4,1970) = 18.160, *p* < 0.001
SSS‐8 [Mean ± SD]	9.97 ± 6.18	7.9 ± 6.00	8.04 ± 5.72	11.58 ± 6.56	9.94 ± 6.74	*F*(4,1970) = 16.581, *p* < 0.001
EQ‐5D [Mean ± SD]	0.72 ± 0.17	0.81 ± 0.18	0.74 ± 0.19	0.65 ± 0.19	0.73 ± 0.17	Kruskal–Wallis *H* = 72.694, *p* < 0.001
Involvement of Psychobehavioral factors (%)	305 (46.7)	9 (2.9)	88 (10.0)	−	6 (12.2)	Pearson's chi‐squared value = 378.68, *p* < 0.001

Abbreviations: AI, acute infection; ANOVA, analysis of variance; EQ‐5D, EuroQol‐5 dimension; FSS, functional somatic syndrome; OD, organic disease; PSYD, psychiatric disorder; SD, standard deviation; SSS‐8, Somatic Symptom Scale‐8; UC, unknown condition.

### 3.3 Clinical features

SSS‐8 score totals were compared between the diagnostic categories, and differences were noted [*F* (4, 1970) = 16.581; *p* < 0.001; Table 1]. Multiple comparisons showed that the SSS‐8 score totals for the FSS and PSYD categories were higher than those for the AI and OD categories, with the UC category showing intermediate scores (Table 2).

**TABLE 2:** Multiple comparison of SSS‐8 total scores

Category I	Category II	Mean difference (I‐II)	SE	*p*	95% CI	
					Lower bound	Higher bound
FSS	AI	2.072	0.415	<0.001	0.910	3.240
	OD	1.929	0.309	<0.001	1.060	2.800
	PSYD	−1.614	0.686	0.188	−3.540	0.320
	UC	0.029	0.886	1.000	−2.460	2.520
AI	OD	−0.143	0.397	1.000	−1.260	0.970
	PSYD	−3.686	0.730	<0.001	−5.740	−1.630
	UC	−2.043	0.921	0.266	−4.630	0.540
OD	PSYD	−3.543	0.676	<0.001	−5.440	−1.640
	UC	−1.900	0.878	0.306	−4.370	0.570
PSYD	UC	1.643	1.071	1.000	−1.370	4.650

Abbreviations: AI, acute infection; CI, confidence interval; FSS, functional somatic syndrome; OD, organic disease; PSYD, psychiatric disorder; SE, standard error; SSS‐8, Somatic Symptom Scale‐8; UC, unknown condition.

EQ‐5D utility values were compared between the diagnostic categories, and differences were found [Kruskal–Wallis H (4) = 72.694; *p* < 0.001; Table 1]. These utility values were the lowest in the PSYD category; intermediate in the FSS, OD, and UC categories; and the highest in the AI category (Table 3).

**TABLE 3:** Multiple comparison of EQ‐5D utility values

Category I	Category II	Mean difference (I‐II)	SE	*p*	95% CI	
					Lower bound	Higher bound
FSS	AI	−0.089	0.012	<0.001	−0.124	−0.054
	OD	−0.020	0.009	0.276	−0.046	0.005
	PSYD	0.070	0.020	0.007	0.012	0.128
	UC	−0.009	0.026	1.000	−0.084	0.065
AI	OD	0.069	0.011	<0.001	0.035	0.102
	PSYD	0.160	0.021	<0.001	0.098	0.221
	UC	0.080	0.027	0.037	0.002	0.158
OD	PSYD	0.090	0.020	<0.001	0.033	0.147
	UC	0.011	0.026	1.000	−0.063	0.085
PSYD	UC	−0.079	0.032	0.132	−0.170	0.010

Abbreviations: AI, acute infection; CI, confidence interval; EQ‐5D, EuroQol‐5 Dimension; FSS, functional somatic syndrome; OD, organic disease; PSYD, psychiatric disorder; SE, standard error; UC, unknown condition.

The effects of psychobehavioral factors on somatic symptoms were compared between diagnostic categories other than PSYD, and differences were noted [*χ* ^2^ value (3) = 378.68; *p* < 0.001; Table 1]. Multiple comparisons showed that the proportion of patients affected by psychobehavioral factors was the highest in the FSS category (46.7% [305 of 653] of patients), with differences noted in comparison with the OD, AI, and UC categories.

## 4 DISCUSSION

This study investigated the characteristics of patients with FSS at a general medicine clinic of a university hospital. FSS occupied one‐third of our study population and was associated with severe somatic symptom burden, moderate HRQoL, and high involvement of psychobehavioral factors.

To the best of our knowledge, this is the first large‐scale study to elucidate the proportion and characteristics of patients with FSS at a general medicine clinic of a university hospital. This diagnostic category has not been widespread until recently, and there are no previous studies that have classified MUS by functional pathology or problem. Therefore, FSS and UC in our data were considered to correspond to MUS in conventional studies. For our study, the combined prevalence of FSS and UC was 36% in general medicine clinic, and in other studies, MUS accounted for 5%–30% of primary care diagnoses^2^ and 37%–68% of specialty care diagnoses.^14^, ^15^ In secondary care settings, each specialty of the clinics is usually related to the characteristic of the patient with MUS. Therefore, the prevalence of FSS in MUS varies according to the settings. As our study population consisted of both primary and secondary healthcare patients, it is suitable that our result was between those reported in the two studies mentioned. Our data revealed that the prevalence of FSS among MUS in general medicine clinic was 93%, indicating the potential usefulness of FSS diagnostic skills in reducing the misdiagnosis of MUS.

Functional somatic syndromes shows high somatic symptom burden rather than demonstrable tissue abnormalities. We still do not have a simple method to diagnose FSS. While the SSS‐8 is a questionnaire to assess somatic symptom burden, our results showed that it was useless as a diagnosis assistance tool to distinguish FSS from UC. FSS such as IBS and FMS are frequently associated with several complications,^22^ while AI and OD are associated with fewer complications. A high total SSS‐8 score may reflect a high incidence of comorbidities. The PSYD category, which comprised patients with psychiatric disorders and excluded patients with SSD and “psychological factors affecting other medical conditions,” showed the highest SSS‐8 score. Gierk et al. have reported that the SSS‐8 score is significantly associated with depression and anxiety,^16^ possibly because the SSS‐8 parameters include scores for sleep disturbance and fatigue (the typical somatic symptoms of depression). Further studies focusing on each item of the SSS‐8 are needed to understand the reason for these inclusions.

The HRQoL status in FSS was approximately the same as that in OD. HRQoL status is reported to be reduced by the same extent for IBS and other chronic organic diseases,^23^ and to decrease more in psychiatric disorders than in physical disorders.^24^ Our results support these findings. In addition, the HRQoL in AI may not have been reduced because of the short morbidity period.

Functional somatic syndromes was associated with high involvement of psychobehavioral factors and differed quite markedly from UC in this respect. In our study, psychobehavioral factors were assessed by a physician only during the first visit, and this might not be sufficient to differentiate between SSD and “psychological factors affecting other medical conditions”, as defined in the DSM‐5. However, the distinction between FSS and SSD is conceptually difficult when somatic symptoms are affected by psychobehavioral factors.^6^ In our study, we did not distinguish these conditions because that was not our aim. Numerous studies have established the involvement of psychobehavioral factors in FSS,^25^, ^26^, ^27^ and our results indicated that the psychosomatic component is one important mechanism of FSS. Thus, in a narrow sense, MUS (corresponding to UC in our data) might be less associated with the involvement of psychobehavioral factors than was supposed.

## 5 LIMITATIONS

First, this study was performed at a single medical institution, and therefore, it might have been subject to selection bias. It is desirable to conduct multicenter studies including general medicine clinics of other university hospitals and community hospitals or primary care offices to validate our findings. Second, the term MUS was not used in the classification, and FSS and UC categories were used instead. However, the definitions of these terms were somewhat vague, and the decision as to whether or not a condition was a functional pathology was partly subjective on the part of the examining physician. We tried to resolve this issue by including three physicians; however, it is uncertain whether this approach was sufficient to eliminate this problem. Autonomic dysfunction, which is considered as one of the mechanisms of FSS,^9^ is expected to be an objective index to distinguish FSS from MUS in the future studies. Third, only the first‐listed diagnosis for each patient was used for classification, and no other comorbid diseases were investigated. It is necessary to evaluate the classification of patients according to the final diagnoses or comorbid diseases.

## 6 CONCLUSIONS

We investigated the characteristics of patients with FSS at a general medicine clinic of a university hospital. FSS comprised one‐third of our study population, and almost all of the supposed MUS (FSS and UC) were diagnosed with FSS. Compared with other organic diseases, FSS showed more severe somatic symptom burden, similar HRQoL, and higher involvement of psychobehavioral factors. It can be useful to improve FSS diagnostic skills for the reduction in MUS misdiagnosis. Psychobehavioral factors might be less associated with MUS (in the narrow sense of the term) than FSS.

## CONFLICT OF INTEREST

The authors declare no conflict of interest.
